# Growth in Children With Noonan Syndrome and Effects of Growth Hormone Treatment on Adult Height

**DOI:** 10.3389/fendo.2021.761171

**Published:** 2021-12-22

**Authors:** Annachiara Libraro, Vito D’Ascanio, Marco Cappa, Mariangela Chiarito, Maria Cristina Digilio, Silvia Einaudi, Anna Grandone, Mohamad Maghnie, Laura Mazzanti, Alessandro Mussa, Giuseppa Patti, Emanuela Scarano, Antonietta Spinuzza, Silvia Vannelli, Malgorzata Gabriela Wasniewska, Giovanni Battista Ferrero, Maria Felicia Faienza

**Affiliations:** ^1^ Department of Biomedical Sciences and Human Oncology, Pediatric Unit, University of Bari “A. Moro”, Bari, Italy; ^2^ National Research Council–Institute of Sciences of Food Production (CNR-ISPA), Bari, Italy; ^3^ Unit of Endocrinology, Bambino Gesù Children’s Hospital, Istituto di Ricovero e Cura a Carattere Scientifico (IRCCS), Rome, Italy; ^4^ Genetics and Rare Diseases Research Division, Bambino Gesù Children’s Hospital, Istituto di Ricovero e Cura a Carattere Scientifico (IRCCS), Rome, Italy; ^5^ Department of Pediatric Endocrinology and Diabetology, Regina Margherita Children Hospital, University of Turin, Turin, Italy; ^6^ Department of Woman, Child and General and Specialized Surgery, University of Campania Luigi Vanvitelli, Naples, Italy; ^7^ Department of Pediatrics, Istituto di Ricovero e Cura a Carattere Scientifico (IRCCS) Istituto Giannina Gaslini, Genova, Italy; ^8^ Department of Neuroscience, Rehabilitation, Ophthalmology, Genetics, Maternal and Child Health–University of Genova, Genoa, Italy; ^9^ Pediatric Rare Diseases Unit, Department of Pediatrics, St. Orsola University Hospital, University of Bologna, Bologna, Italy; ^10^ Department of Public Health and Pediatrics, University of Torino, Torino, Italy; ^11^ Department of Human Pathology of Adulthood and Childhood Gaetano Barresi, Gaetano Martino University Hospital, University of Messina, Messina, Italy; ^12^ Department of Clinical and Biological Sciences, University of Torino, Torino, Italy

**Keywords:** Noonan Syndrome, growth, children, growth hormone treatment, adult height

## Abstract

**Objectives:**

Growth impairment is a common manifestation in Noonan syndrome (NS). Recombinant human GH (rhGH) treatment has been shown to increase growth and adult height (AH) in a few studies. We aimed to evaluate the growth trajectory towards the AH, and the effects of rhGH treatment in a large cohort of NS children.

**Methods:**

Retrospective, multicenter, cohort study including subjects with genetic diagnosis of NS. A total of 228 NS patients, 154 with *PTPN11* mutations, 94 who reached AH, were recruited. Auxological data were collected at 2, 5, and 10 years, at pubertal onset, at AH. Sixty-eight NS subjects affected with GH deficiency (GHD) were treated with rhGH at a mean dose of 0.24 mg/kg per week until AH achievement.

**Results:**

ANOVA analysis showed a significant difference between birth length and height standard deviation scores (HSDS) at the different key ages (*p<0.001*), while no significant differences were found between HSDS measurements at 2, 5, and 10 years, at pubertal onset, and at AH. HSDS increased from −3.10 ± 0.84 to −2.31 ± 0.99 during rhGH treatment, with a total height gain of 0.79 ± 0.74, and no significant difference between untreated and treated NS at AH.

**Conclusions:**

rhGH treatment at the standard dose used for children with GH idiopathic deficiency is effective in improving growth and AH in NS with GHD. Further studies are needed to assess genotype-specific response to rhGH treatment in the different pathogenic variants of *PTPN11* gene and in the less common genotypes.

## Introduction

Noonan syndrome (NS) is a multisystem disorder characterized by facial and skeletal dysmorphisms, short stature, congenital heart diseases, organ dysfunction, and mild-to-moderate developmental/learning delay (1, 2). NS can be considered as a phenotypic spectrum in the context of Rasopathies which represent genetically heterogeneous diseases due to mutations in genes involved in the RAS-MAPK signaling cascade (3, 4). Several genes have been associated with NS, covering the majority of cases (5–11), although the diagnosis remains clinical in 20–30% of subjects. Short stature is a common feature in NS subjects, and adult height (AH) is variably affected (12–17). Birth weight (BW) and birth length (BL) are usually within the normal range, and the growth failure generally develops during the first year of life. Afterwards, the decline of growth trajectory becomes evident because of delayed puberty and reduced pubertal growth spurt (12, 13, 18). The mean AH is about −2 standard deviation scores (SDS) compared to normal population (18). The effects of recombinant human GH (rhGH) treatment in NS subjects are still debated (19–24). Long-term rhGH therapy ranging from 4.2 to 11.8 years determines a normalization of AH as for Ranke standards, with height gains varying from 0.6 to 2.0 SDS (19, 20); however, NS patients do not show the typical catch-up growth of subjects with isolated growth hormone deficiency (GHD) (25). The response to rhGH treatment in NS can be affected by several factors, such as age at the treatment start, genotype, dose, and treatment duration (26).

Randomized studies in NS patients who reached AH have not been published so far, and available data are difficult to compare due to the heterogeneous treatment protocols, sample size, as well as different cohort selection criteria and limited data on AH.

The aims of this multicenter retrospective study were to evaluate the growth trajectory towards the AH, and the effects of rhGH treatment in a large cohort of Italian NS children, by comparing rhGH-treated *versus* untreated NS subjects.

## Methods

### Subjects

Seven Italian Pediatric Endocrinology Centers participated in this retrospective study. Inclusion criteria were molecular diagnosis of NS and at least three height measurements among the key ages recorded on standard datasheets (2, 5, and 10 years, at pubertal onset, and at AH), in order to evaluate growth trajectory. For subjects who had undergone rhGH therapy, height measurements expressed as height SDS (HSDS), were evaluated at the beginning of treatment, after the first and the second year, and at the end of treatment. Anthropometric measurements were compared with the standard growth charts for the Italian population (27, 28) and for NS (12) and were expressed as SDS. Body mass index (BMI) was calculated as the ratio weight/height^2^. Bone age (BA) was determined using the Greulich and Pyle method and evaluated for each key age recorded on standard datasheets.

Patients with height (H) ≤ −3 SDS, or H ≤ −2 SDS and growth velocity (GV) ≤ −1 SDS, or severe reduction in GV (≤ −2 SDS/year) were tested for GHD by pharmacological stimulation tests, as indicated by the Italian Drug Agency (Agenzia Italiana del Farmaco, AIFA), note number 39. GHD was defined as a peak of GH value less than 10 ng/ml or 8 ng/ml, according to revised criteria starting from 2015, in two GH stimulating tests.

Pubertal stages were assessed with the Tanner method (29). GV (cm/year) was obtained at every time point as the ratio between the difference in height and the difference in age with respect to the previous time point, and evaluated on Tanner charts (30). AH was defined as the height measured when GV was below 1 cm/year and when epiphyseal closure occurred on wrist radiograph. Target height (TH) was calculated as mid-parental height plus 6.5 cm for males and minus 6.5 cm for females.

The study was approved by the Comitato Etico Indipendente, Azienda Ospedaliero-Universitaria “Consorziale Policlinico,” Bari, Italy. Children’s parents or guardians gave written informed consent.

### Statistical Analysis

Numerical data considered in the study were expressed as mean ± SDS. Mean HSDS for NS population was compared with those of the normal population (28) and those of disease-specific reference population (12). Student’s test (or one sample *t-*test) was used for normally distributed data; Mann–Whitney U test was used for not normally distributed data. ANOVA was applied to compare means between more than two groups. The level of significance was set in all cases at *p*<0.05. The normal distribution of the measured variables was assessed by Shapiro-Wilk test, whereas the homogeneity of variances across groups was verified using Levene’s test. Variables with non-normal distribution were expressed as medians and interquartile ranges (IQRs) and were compared using Kruskal–Wallis test. In case of significant results, pairwise comparisons using Tukey test or Dunn’s method for parametric or non-parametric ANOVA analysis were performed, respectively. Friedman test (ANOVA on rank for repeated measurements) was used to evaluate the effect of rhGH therapy on height measurements during the treatment period. Multiple regression analysis, using the forward stepwise approach, was used to study the effect of some variables on the outcome, represented by the total statural gain ∆HSDS (height at the end of therapy − height at baseline). Normality test (p>0.05), equal variance test (p>0.05), and Durbin-Watson test (value between 1.5 and 2.5) were verified before performing multiple regression analysis, to assess normal distribution of population, variance homogeneity, and autocorrelation in the residuals, respectively. Statistical analyses were performed using SigmaPlot^®^ Software, version 12 for Windows.

## Results

**Figure 1:**
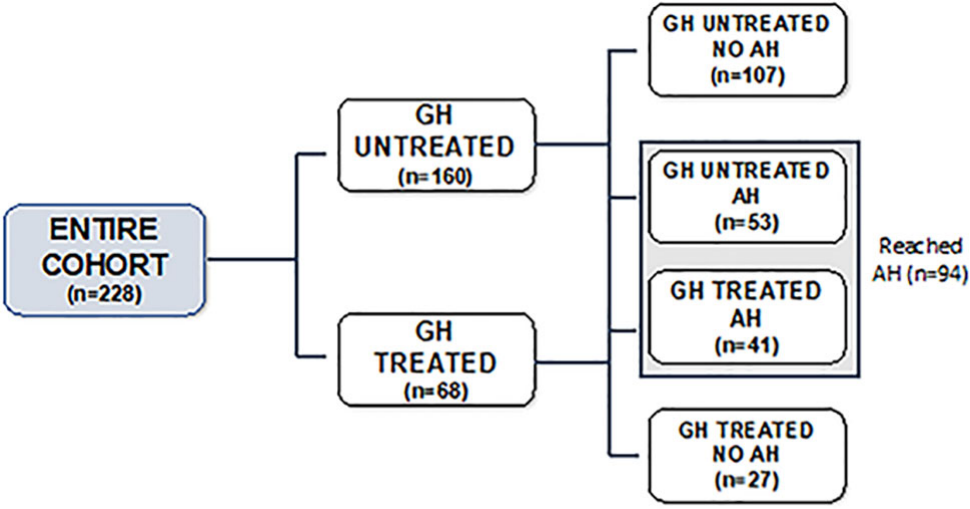
Flow chart of the study cohort.

Data from 228 patients with molecular diagnosis of NS (132 males) were collected. All cases were *de novo* mutations. Among them, 154 (67.5%) had *PTPN11* mutations, while 74 (32.5%) had the following gene mutations: 21 *SOS1*, 16 *SHOC2*, 9 *RAF1*, 8 *KRAS*, 5 *BRAF*, 6 *LZTR1*, 3 *RIT1*, 2 *SOS2*, 1 *MEK*, 1 *NRAS*, 1 *CBL*, and 1 *MAP2K2*. Most of the patients (196 out of 228; 86%) were born at term; 32 (14%) were born before 37 weeks. Overall, 160 patients (70.2%) had cardiac malformations, and 18 (11.2%) underwent surgery. Data on AH were obtained from 94 patients (41.2%) (**Figure 1**). A group of 68 NS patients (40 males), resulted having GHD (mean GH peak 7.07 + 3.67 ng/ml, <7 ng/ml in 67% and <5 ng/ml in 36% of the NS subjects) was treated with rhGH at a mean dose of 0.24 mg/kg per week until the AH.

### Growth Trend of Patients’ Cohort

NS patients were significantly shorter (*p=0.008*) and had a bigger head circumference (*p= 0.001)* at birth, while BW was similar to healthy neonates (**Table 1**). HSDS measurements of patients evaluated at 2, 5, 10 years, at pubertal onset, and at AH were −2.18 ± 1.00, −2.22 ± 0.96, −2.01 ± 0.89, −2.27 ± 0.99 and −2.05 ± 1.01, respectively, according to Cacciari growth charts, and 0.26 ± 1.23, 0.17 ± 1.09, 0.20 ± 0.97, −0.03 ± 1.08, and 0.26 ± 0.89, respectively, according to Ranke standard.

**Table 1:** Auxological parameters of the entire population at birth.

	Mean	SD
Gestational age (weeks)	38.2	2.4
Birth weight (kg)	3.250	0.50
Birth weight (SDS)	0.04°	1.30
Birth length (cm)	49.4	2.48
Birth length (SDS)	−0.35^*^	1.33
Head circumference (SDS)	0.50^*^	1.34
TH SDS	−0.62^*^	1.01

Data are shown as mean ± SD. TH, target height; SDS, standard deviation scores.

*Statistically significant difference between NS patients and healthy neonates (birth length SDS p=0.008; head circumference SDS p=0.001; and TH SDS p<0.001; °no statistically significant difference.

**Figure 2:**
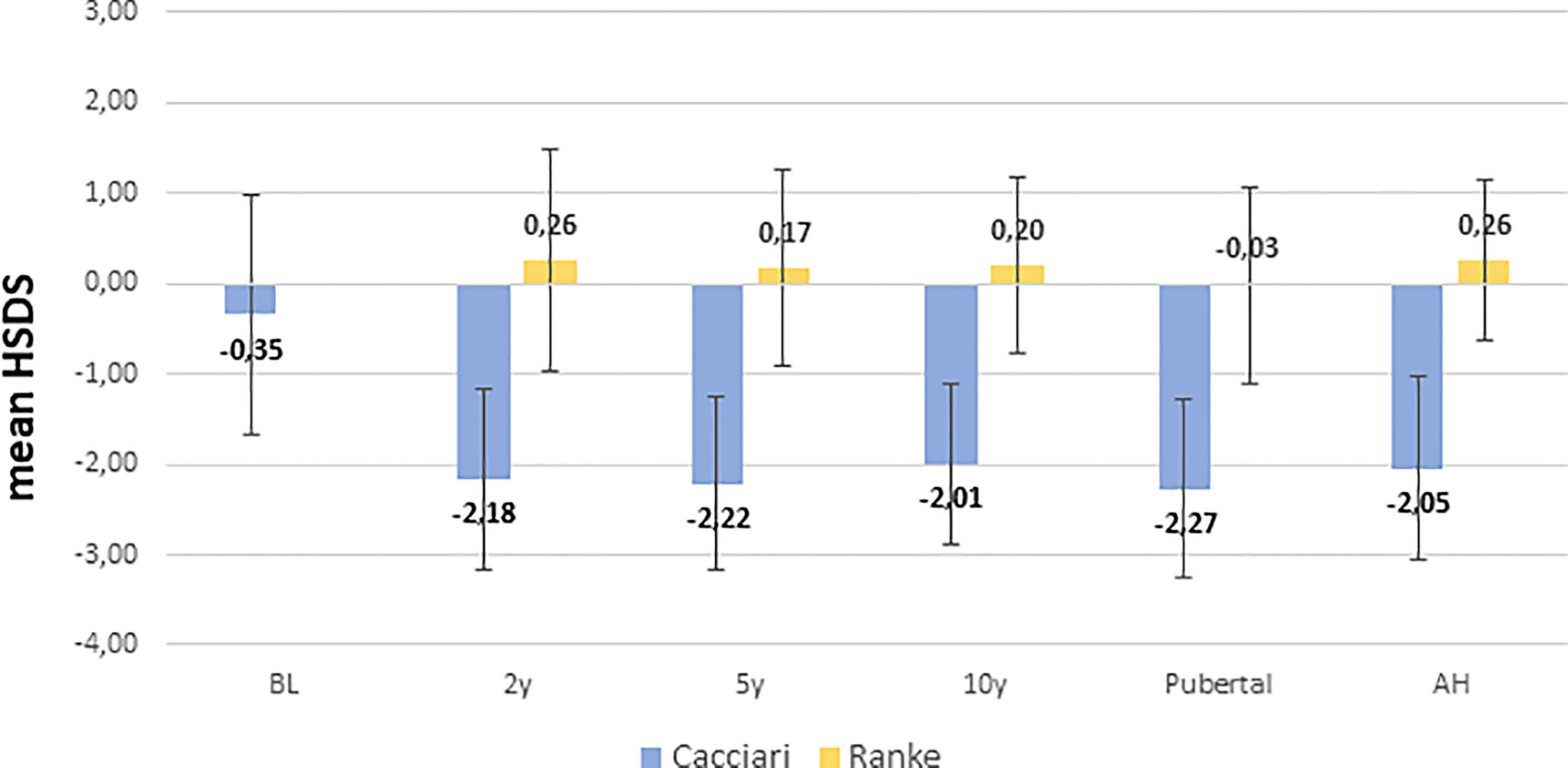
Height SDS from birth to AH according to Cacciari and Ranke standard. BL, birth length; H: HSDS measured at 2, 5, 10 years, at pubertal onset, and at adult height (AH).

ANOVA analysis among length/height measurements showed a significant difference between BL SDS and HSDS at the different key ages (*p<0.001*), while no significant differences were found between HSDS measurements at 2, 5, and 10 years, at pubertal onset, and at AH, as shown in **Figure 2**. A similar trend was found for weight SDS (WSDS) at the different key ages: 0.04 ± 1.30 at birth, −1.92 ± 1.27, −1.79 ± 0.97, −1.72 ± 0.98, and −2.01 ± 1.19 at 2, 5, and 10 years, at pubertal onset, respectively, and −1.70 ± 1.49 at AH.

### Growth Trend According to the Genotype

Patients were divided into two groups according to genotype: *PTPN11+* (n= 154; 67.5%) and *PTPN11*−, the latter including patients with mutation in the other NS-related genes. BL did not differ between the two groups, while *PTPN11*+ subjects had a lower BW than *PTPN11*− (mean value −0.24 *versus* 0.63 SDS, respectively; *p<0.001*). At the other crucial time points, no significant difference was found between the two genotype groups.

### Growth Trend According to the Sex

At the onset of puberty, NS females had a mean age of 12.1 ± 2.3 years, with a mean BA of 11.3 ± 1.7 years, while male patients had a mean age of 12.1 ± 1.3 years, with a mean BA of 10.8 ± 1.3 years. The frequency of delayed puberty was 45% in females and 10% in males.

In females, HSDS decreased at puberty onset (−2.47 ± 1.15 SDS; 0.32 ± 1.36 Ranke standard) and increased during the pubertal spurt, reaching an AH SDS of −2.02 ± 1.09 (−0.04 ± 0.94 Ranke standard). In boys, a steadier growth trend with a HSDS of −2.14 ± 0.87 (0.02 ± 0.96 Ranke standard) at pubertal onset and −2.08 ± 0.96 SDS (0.23 ± 0.85 Ranke standard) at AH was observed. According to genotype, no significant difference was found between height at the start of puberty in *PTPN11*+ and *PTPN11*− subjects.

### Growth Trend of rhGH-Treated NS Subjects

Among rhGH-treated NS subjects, 48 (71%) were *PTPN11+*, while the other 20 subjects (29%) showed different genotypes (9 *SCHOC2*, 2 *RAF1*, 3 *SOS1*, 4 *KRAS*, 2 *BRAF*). At the beginning of treatment, the mean age was 7.2 ± 3.5 years (range: 0.9–14.1 years). The mean duration of rhGH therapy was 6.3 ± 4.0 years, and the mean dose of rhGH administered was 0.24 mg/kg per week. HSDS evaluated at the beginning of treatment, after the first and the second year, and at the end of treatment is shown in **Table 2**. The height gain (ΔH) SDS, defined as the difference between the height at the end minus height at the start of rhGH treatment, was 0.79 ± 0.74. and 1.35 ± 0.36, according to general population and Ranke standard, respectively.

**Table 2:** Auxological features and IGF-1 levels of NS subjects during rhGH treatment.

	Start	1-y	2-y	End	p-value
**HSDS** (28)	−3.10^a^ ± 0.84	−2.66^b^ ± 0.86	−2.48^bc^ ± 0.90	−2.3^c^ ± 0.99	***<0.001***
**HSDS** (12)	−0.90^a^ ± 0.92	−0.39^b^ ± 0.94	−0.34^b^ ± 0.99	0.45^c^ ± 1.28	***<0.001***
**HSDS female** (28)	−3.49^a^ ± 0.78	−2.97^ab^ ± 0.94	−2.74^bc^ ± 1.03	−2.33^c^ ± 1.22	***<0.001***
**HSDS female** (12)	−1.02^a^ ± 0.84	−0.53^b^ ± 1.03	−0.4^b^ ± 1.12	−0.01^c^ ± 1.16	***<0.001***
**HSDS male** (28)	−2.68^a^ ± 0.72	−2.31^b^ ± 0.75	−2.18^b^ ± 0.80	−2.15^b^ ± 0.92	***<0.001***
**HSDS male** (12)	−0.20^a^ ± 0.80	0.23^b^ ± 0.83	0.06^b^ ± 0.88	0.72^c^ ± 1.18	***<0.001***
**BA (yrs)**	5.80^a^ ± 3.81	6.59^ab^ ± 3.76	8.36^b^ ± 3.70	–	***0.012***
**BA/chronological age**	0.72^a^ ± 0.19	0.77^b^ ± 0.16	0.84^b^ ± 0.15	–	***0.021***
**IGF-1 (ng/ml)**	82^a^ ± 50	185^b^ ± 114	213^b^ ± 124	370^c^ ± 189	***<0.001***
**IGF-1 SDS**	−1.25^a^ ± 1.50	0.35^b^ ± 1.69	0.50^b^ ± 1.54	0.66^b^ ± 2.12	***<0.001***

For each parameter, a comparison among groups (time points) by ANOVA was made. Data are shown as mean ± SD and expressed as standard deviation scores (SDS). Values with different superscript letters had a statistically significant difference; values with a superscript letter in common were statistically equivalent.

HSDS was also evaluated in male and female patients (**Table 2**). As for BMI, no significant difference between the beginning and the end of treatment was found (−0.68 ± 1.24 *versus* −0.83 ± 1.46, respectively; *p=0.917*). There was no significant difference in age and weight between male and female patients at the beginning of treatment, but female patients were significantly shorter (−3.37 ± 0.74 SDS; −0.93± 0.79 Ranke Standard) than male patients (−2.80 ± 0.60 SDS; −0.38 ± 0.66 Ranke Standard) (p=0.001).

**Figure 3:**
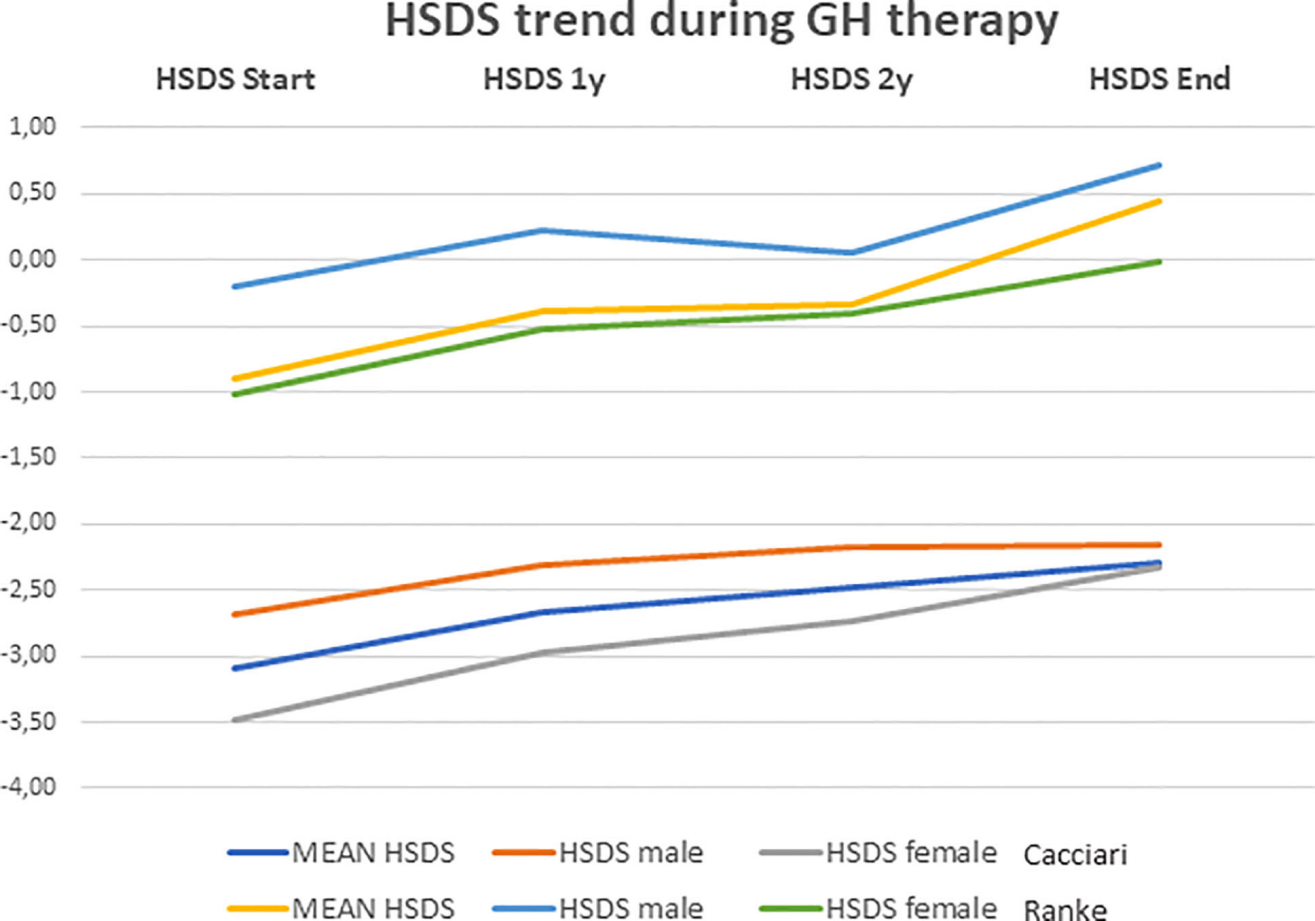
Height growth trend recorded during rhGH therapy. Data are shown as mean ± SD and expressed as standard deviation scores (SDS).

Multiple comparisons performed using Tukey test showed that there was an increase in HSDS mean values during rhGH treatment with the best results obtained in the first year (**Table 2**). Indeed, from the first year to the second one, there were not significant differences in HSDS mean values, as well as from the second year to the end of therapy (*p>0.05*). The trend of HSDS during the treatment period is shown in **Figure 3**. Serum IGF-I levels significantly increased from the beginning to the end of treatment (*p<0.001*) (**Table 2**). rhGH dose decreased from baseline (0.24 ± 0.04 mg/kg per week) to the end of therapy (0.21 ± 0.05 mg/kg per week) (*p=0.011*). BA increased from 5.80 ± 3.81 years to 6.59 ± 3.76 years after one year, and to 8.36 ± 3.70 years after two years of treatment (*p=0.012*). The BA/chronologic age ratio increased from 0.72 ± 0.19 at the start of treatment to 0.77 ± 0.16 after one year, and to 0.84 ± 0.15 after 2 years of treatment (*p=0.021*). AH was reached by 41 out of the 68 NS subjects (60.3%) who had undergone rhGH treatment, at the mean age of 19.42 ± 1.38 years, and the mean value was −2.24 ± 0.89 SDS (0.09 ± 0.79 Ranke standard).

### Comparison of Growth Trajectory and AH Between rhGH-Treated and Untreated NS Subjects

The comparison of H measurements between the rhGH treated and untreated NS subjects showed a significant difference between the 2-year-olds groups (*p=0.024*), the 5-year-olds groups (*p<0.001*), the pubertal onset groups (*p <0.001*), and a slightly significant difference between the 10-year-olds groups (*p=0.043*). On the contrary, no significant difference was found between the AH groups (*p=0.123*) (**Table 3**). Nevertheless, treated NS patients as well as untreated NS patients were not able to reach their TH SDS: −0.89 ± 1.00 (1.28 ± 0.88 Ranke standard) and −0.41 ± 0.99 (1.70 ± 0.86 Ranke standard), respectively. Indeed, a comparison between TH and AH of both NS treated and untreated patients showed a significant difference (*p<0.001*) (**Table 3**).

**Table 3:** HSDS in NS treated and untreated subjects.

HSDS	2 years old	5 years old	10 years old	Pubertal onset	Adult height	TH
**rhGH treated**	−2.57 ± 0.95 (−0.72 ± 1.17)	−2.66 ± 0.82 (−0.65 ± 1.00)	−2.29 ± 0.87 (−0.1 ± 0.95)	−2.65 ± 1.05 (−0.52 ± 1.12)	−2.24 ± 0.89 (0.09 ± 0.79)	−0.89 ± 1.00 (1.28 ± 0.88)
**Untreated**	−1.85 ± 1.02 (0.17 ± 1.25)	−1.80 ± 0.94 (0.32 ± 1.07)	−1.94 ± 0.89 (0.28 ± 0.98)	−1.84 ± 0.84 (0.37 ± 0.94)	−1.88 ± 1.10 (0.41 ± 0.97)	−0.41 ± 0.99 (1.70 ± 0.86)
***p value***	***0.024***	***<0.001***	***0.043***	***<0.001***	***0.123***	p<0.001*

Data are shown as mean ± SD and expressed as Cacciari SDS or Ranke SDS in brackets. * refers to the comparison between AH and TH for treated and untreated patients.

### Parameters Affecting ∆HSDS During rhGH Treatment

To assess which parameters affected ∆HSDS during rhGH therapy, a multiple regression analysis was performed. ∆HSDS was set as the outcome (dependent variable), while chronological age, IGF-I, rhGH dose, baseline HSDS, type of mutation, TH, and sex were considered as independent variables. To perform this analysis, qualitative variables such as type of mutation and sex were transformed into dummy variables (values 0 or 1). In particular, value 1 was assigned to *PTPN11+* subset and to male sex patients’ subgroup. Taking into account pubertal age of NS patients, two different kinds of multiple regression analysis were performed.

**Table 4** shows data of the first regression model regarding prepubertal NS patients. In this case, the outcome ∆HSDS can be predicted from a linear combination of the following variables: sex (*R^2^ = 0.431, p<0.001*), IGF-I (*R^2^ = 0.758, p<0.001*), and type of mutation (*R^2^ = 0.956, p<0.001*). These results showed that patients with female sex, *PTPN11*+, and lower IGF-I values were more likely to achieve a wider ∆HSDS, hence a higher height at the end of therapy.

**Table 4:** Multiple regression data for prepubertal and pubertal subjects.

Prepubertal multivariate analysis	β	R^2^	*p value*
**Sex**	−1.13 ± 0.83	0.431	***<0.001***
**IGF-I baseline**	−0.01 ± 0.52	0.758	***<0.001***
**Mutation**	0.61 ± 0.45	0.956	***<0.001***
Normality test			Passed p=0.877
Costant variance test			Passed p=0.353
Durbin-Watson statistic			Passed 1.650
**Pubertal multivariate analysis**	**β**	**R^2^**	***p value***
**Mutation**	2.40 ± 0.71	0.281	***0.008***
**Sex**	1.12 ± 0.48	0.482	***0.049***
Normality test			Passed p=0.291
Costant variance test			Passed p=0.054
Durbin-Watson statistic			Passed 2.268

Regression coefficients (β) were expressed as mean ± SD. Coefficient of determination (R^2^) was calculated for each independent variable that showed a statistic significance (p<0.05).

The second regression model evaluated NS pubertal patients in order to estimate their tendency to reach TH. In this model, the difference between HSDS at the end of treatment and TH was set as the outcome, while chronological age, IGF-I, rhGH dose, and HSDS measured at baseline, type of mutation, and sex were considered as independent variables. In this model, it was found that the dependent variable can be predicted from a linear combination of mutation (*R^2^ = 0.281*, *p=0.008*) and sex (*R^2^ = 0.482*, *p=0.052*). These results suggested that males and patients *PTPN11+* reached a height at the end of therapy closer to their TH values (**Table 4**).

## Discussion

We analyzed the growth pattern of a large cohort of NS children from birth towards AH, and we compared rhGH-treated NS patients *versus* untreated ones.

Our cohort of NS subjects had a significantly shorter BL than healthy neonates. Furthermore, *PTPN11*+ subjects had a more evident growth failure at birth than those carrying other gene mutations, in agreement with previous studies (17, 18).

The postnatal growth in our cohort showed a significant difference between BL SDS and HSDS at the different key ages, while no differences in terms of HSDS were found starting from the age of 2 until AH. An early impairment of height within the first 2 years of life of about −1.83 SDS, followed by a steadiness of the growth trend around −2.0 SDS until the age of 10 years, appears to be an intrinsic feature of NS subjects, as previously reported (15, 31). According to the genotype, we did not find any difference in height and weight between *PTPN11* + and *PTPN11*− subjects at the key time points analyzed.

About 30% of our NS subjects had GHD in agreement with other reports (22, 25). The data obtained from our cohort showed the effectiveness of rhGH therapy in normalizing the AH according to Ranke standard but not for the general standard. Therefore, at the end of therapy, the NS-treated group had a stature comparable to that of the untreated ones, even though both groups were not able to reach their TH. Similar results have been reported by Tamburrino et al. in a cohort of patients affected by RASopathies (25). Furthermore, Seok et al. demonstrated an increase of HSDS and GV after 3 years of treatment in prepubertal NS children, with a similar response between NS and GHD patients (32). Malaquias et al. in a cohort of NS *PTPN11* + patients demonstrated the benefits of rhGh therapy on AH, compared with a small cohort of *PTPN11*− subjects (33).

There are a few studies that have reported long-term results on the efficacy of rhGH treatment in NS (34–36). Recently, in two complementary non-interventional studies, NordiNet^®^ IOS and ANSWER, it has been demonstrated that NS children treated with rhGH achieved significant height gain during the first 3 years of follow-up (37).

Regarding the height gain, our NS patients showed the best response during the first year of rhGH treatment, in agreement with previous studies (19–22, 24, 34, 38). In addition to this finding, Ranke et al. found a close relationship between near AH and the first-year response, suggesting an algorithm predicting height velocity during the first year of treatment in order to have the best long-term outcome with the optimization of the therapy (39).

When comparing the growth trend between females and males in response to rhGH treatment, although NS females displayed a greater pretherapy growth impairment than males, they recovered better than males after rhGH therapy.

Regarding the dose of rhGH, no significant differences were observed in our NS patients, possibly due to the low variability of the doses used. In the study of Osio et al. (20), no beneficial effect on AH was found in subjects treated at the highest dosage (0.66 *vs* 0.33 μg/kg/die). In contrast, a significantly greater increase in HSDS has been observed with 0.066 *vs* 0.033 mg/kg/day of rhGH without adverse effects (40, 41). The multiple regression analysis highlighted the crucial role of starting therapy before puberty; indeed, the time of therapy during prepuberty is one of the factors most closely related to the height gain.

The genotypic heterogeneity underlying NS represents a further factor that can explain the individual variability of response to rhGH treatment (24, 36, 42). Indeed, in our study we found that mutations in *PTPN11* correlate positively with height gain in patients starting therapy in prepuberty, and with the tendency to reach their TH in patients starting it after puberty.

A limitation of the study is that our cohort was treated with a dose of rhGH in the range of GHD and we do not know if the use of a higher dose of rhGH would have led to a better response in terms of AH.

## Conclusion

The main findings of this study are the following: (i) rhGH treatment in GH deficient NS subjects is effective in improving height gain and AH, according to Ranke standard; (ii) the greatest loss in height SDS occurs within the first 2 years of life in NS subjects, thus it is recommended to start rhGH treatment early; (iii) GH deficiency alone is insufficient to explain the cause of short stature in NS subjects.

More studies are required to investigate which genotype or molecular alteration produces a good response to the treatment in order to more accurately select the candidates for rhGH therapy.

## Data Availability Statement

The original contributions presented in the study are included in the article/supplementary material. Further inquiries can be directed to the corresponding author.

## Ethics Statement

The studies involving human participants were reviewed and approved by Comitato Etico Indipendente, Azienda Ospedaliero-Universitaria “Consorziale Policlinico,” Bari, Italy. Written informed consent to participate in this study was provided by the participants’ legal guardian/next of kin.

## Author Contributions

AL and MF conceptualized and designed the study, drafted the initial manuscript, and reviewed and revised the manuscript. VD’A performed statistical analysis. MCh, MD, AG, SE, AM, GP, ES, AS, and SV collected data and rewieved the manuscript. MCa, MM, LM, MW, and GF coordinated and supervised data collection and critically reviewed the manuscript for important intellectual content. All authors contributed to the article and approved the submitted version. All authors agree to be accountable for all aspects of the work.

## Conflict of Interest

The authors declare that the research was conducted in the absence of any commercial or financial relationships that could be construed as a potential conflict of interest.

## Publisher’s Note

All claims expressed in this article are solely those of the authors and do not necessarily represent those of their affiliated organizations, or those of the publisher, the editors and the reviewers. Any product that may be evaluated in this article, or claim that may be made by its manufacturer, is not guaranteed or endorsed by the publisher.

## Abbreviations

NS, Noonan syndrome; AH, adult height; GH, growth hormone; rhGH, recombinant human GH; GHD, GH deficiency; HSDS, height standard deviation scores; SDS, standard deviation scores; BA, bone age; BW, birth weight; BL, birth length; BMI, body mass index; GV, growth velocity; TH, target height; ΔH, height gain; IQRs, interquartile ranges; ∆HSDS, height at the end of therapy − height at baseline; WSDS, weight SDS.
